# HIV-gp140-Specific Antibodies Generated From Indian Long-Term Non-Progressors Mediate Potent ADCC Activity and Effectively Lyse Reactivated HIV Reservoir

**DOI:** 10.3389/fimmu.2022.844610

**Published:** 2022-03-02

**Authors:** Jayshree R. Dhande, Rajani D. Bagul, Madhuri R. Thakar

**Affiliations:** ICMR-National AIDS Research Institute, Pune, India

**Keywords:** antibody-dependent cellular cytotoxicity (ADCC), reactivated latent reservoirs, HIV-gp140 antibodies, long-term non-progressors, HIV-specific memory B cells

## Abstract

Strategies to reduce the human immunodeficiency virus (HIV) reservoir are urgently required. The antibody-dependent cellular cytotoxicity (ADCC)-mediating anti-HIV antibodies have shown an association with HIV control. We assessed if such antibodies can be generated *in vitro* and whether the generated antibodies can facilitate the reduction of reactivated HIV reservoir. We isolated HIV-1-gp140-specific memory B cells from HIV-1-infected long-term non-progressors (LTNPs) with or without plasma ADCC and cultured them to generate anti-HIV antibodies. The ability of the generated antibodies to mediate ADCC and facilitate NK cell-mediated lysis of reactivated HIV reservoir was assessed by the rapid fluorometric antibody-dependent cellular cytotoxicity assay and a flow-based novel latency reduction assay, respectively. All LTNPs showed the presence of gp140-specific memory B cells [median: 0.79% (0.54%–1.225%)], which were successfully differentiated into plasma cells [median 72.0% (68.7–82.2%)] in an *in-vitro* culture and secreted antibodies [median OD: 0.253 (0.205–0.274)]. The HIV-gp140-specific antibodies were generated from 11/13 LTNPs irrespective of their plasma ADCC status. The generated antibodies from LTNPs with plasma ADCC showed higher ADCC potency (median: 37.6%, IQR: 32.95%–51%) and higher reduction in reactivated HIV reservoir (median: 62.5%, IQR: 58.71%–64.92%) as compared with the antibodies generated from LTNPs without plasma ADCC (ADCC: median: 8.85%, IQR: 8%–9.7%; and % p24 reduction median: 13.84, IQR: 9.863%–17.81%). The potency of these antibodies to reduce latent reservoir was two-fold higher than the respective plasma ADCC. The study showed that the potent ADCC-mediating antibodies could be generated from memory B cells of the LTNPs with plasma ADCC activity. These antibodies also showed potent ability to facilitate NK cell-mediated lysis of reactivated HIV reservoirs. It also indicated that memory B cells from individuals with plasma ADCC activity should be preferentially used for such antibody generation. The important role of these antibodies in the reduction of latent reservoirs needs to be further evaluated as a useful strategy to obtain a functional cure for HIV infection.

## Introduction

In human immunodeficiency virus (HIV) infection, strategies to reduce the cellular reservoir of HIV are urgently required. At present, a shock and kill approach is being studied which involves the reactivation of the reservoir (shock) and killing the same by the anti-HIV immune response. For such reduction, the HIV-specific broadly neutralizing monoclonal antibodies generated from the memory B cells of HIV-infected individuals are being tested in a clinical trial (1–3) but until now showed only transient suppression of viral rebound under antiretroviral treatment interruptions (4, 5). Hence, the effect of the neutralizing monoclonal antibodies on the size of HIV reservoirs is unclear.

The antibodies can also destroy the infected cells by activating the effector cells such as natural killer (NK) cells through the Fc–FcR interaction. The process is referred to as antibody-dependent cellular cytotoxicity (ADCC). The ADCC has shown association with HIV control (6, 7) and protection (8–10). Also, the potent monoclonal antibodies generated against the conformational epitope (A32) of the C1 region of HIV-1 Env gp120 have been reported to mediate ADCC (11). The monoclonal antibodies isolated from HIV controllers have been shown to mediate ADCC and other Fc functions (7, 12). The anti-HIV neutralizing monoclonal antibodies also have shown lysis of the infected cells *via* ADCC (13). Hence, it was proposed that the ADCC-mediating anti-HIV antibodies could also reduce the latent reservoir by facilitating NK cell-mediated killing of the reactivated reservoirs (14, 15). In our previous study, we have shown that the antibodies purified from the plasma of long-term non-progressors (LTNPs) can mediate lysis of the Env-reactivated resting memory CD4+ T cells (16). All these studies indicated a possible role of ADCC-mediating antibodies in reducing latent reservoir.

However, it is known that the long-term ART reduced ADCC activity (17), making it difficult to depend upon the ADCC activity of plasma antibodies for lysis of reactivated reservoir. Also, the memory B-cell compartment is compromised in HIV infection (18–20) leading to a defective antibody response. These observations indicate that the passive transfer of such antibodies generated from HIV-infected individuals with functional memory B cells would be a better choice. Our previous study has shown functional memory B-cell compartment in Indian LTNPs.

Considering the available information, we conducted the present study to answer two questions: 1) Whether memory B cells from LTNPs could differentiate into plasma cells and generate HIV-specific antibodies, and 2) if so, whether these antibodies can mediate ADCC activity and facilitate natural killer (NK) cell-mediated lysis of reactivated HIV reservoirs. The LTNPs from the institutional cohort showing the presence or absence of ADCC-mediating antibodies were enrolled in the study.

We showed that the HIV-gp140-specific memory B cells sorted from the LTNPs were successfully differentiated into plasma cells in a 10-day culture and secreted anti-gp140-specific antibodies. These antibodies showed the ability to mediate ADCC and lyse the Env-reactivated latently infected resting memory CD4+ T cells *via* NK cells. We also observed that the HIV-specific memory B cells from LTNPs showing plasma ADCC activity could generate antibodies with higher ADCC potency. These antibodies also have a higher potential to lyse reactivated latent reservoirs.

## Methods

**Figure 1:**
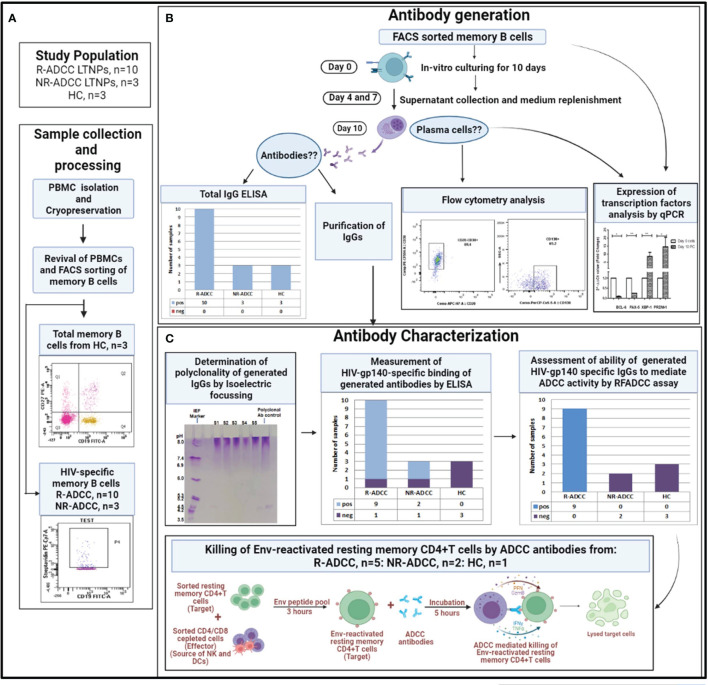
Summary of the experimental workflow of the study. **(A)** Whole blood samples were collected from long-term non-progressors (LTNPs) with and without plasma antibody-dependent cellular cytotoxicity (ADCC) activity (R-ADCC, *n* = 10 and NR-ADCC, *n* = 3, respectively) and 3 healthy individuals (HCs) and processed for the separation and cryopreservation of peripheral blood mononuclear cells (PBMCs). The PBMCs were then revived and memory B cells from three HCs and HIV-specific memory B cells from R-ADCC (*n* = 10) and NR-ADCC LTNPs (*n* = 3) were FACS sorted. **(B)** Antibody generation: the FACS-sorted memory B cells from all the samples were cultured for 10 days. On day 10, supernatants were assessed for the presence of antibodies and were then column purified. The cells were assessed for the differentiation into plasma cells by flow cytometry and real-time PCR analysis at day 10. **(C)** Antibody characterization: the generated antibodies were then assessed for the presence of gp140 antibodies using ELISA, their ability to mediate ADCC using rapid fluorometric antibody-dependent cellular cytotoxicity (RF-ADCC) assay, and their ability to facilitate NK cell-mediated lysis of Env-reactivated latent reservoirs using a flow-based latency reduction assay as reported earlier (16).

The experimental workflow for the generation and characterization of HIV-specific polyclonal antibodies is summarized in **Figure 1**.

### Study Participants

Thirteen [3 men/10 women; age 35 years (30.5–41.5) years] LTNPs [HIV-infected individuals who are asymptomatic and maintained a stable CD4 count of ≥500 cells/mm^3^ for a period of 7 or more years without antiretroviral therapy (ART)] (21) (viral load: median: 3 log copies/ml; IQR: 2.6–4 log copies/ml) were enrolled from the outpatient clinic of the institute. Based on their plasma ADCC activity observed earlier (7), they were categorized as R-ADCC (*n* = 10, showed anti-HIV-1 C Env ADCC) and NR-ADCC (*n* = 3, did not show ADCC against any HIV-1 antigen) LTNPs (7). Three (2 men/1 woman) [age: 32 years (27–34 years)] HIV-seronegative controls (HCs) were also enrolled to assess the generation of non-specific antibodies. Additionally, eight HIV-infected ART-naive individuals (stable CD4 count ≥500 cells/mm^3^ at least for 3 years) were enrolled as a source of HIV reservoir and also the effector cells in latent reservoir reduction assay.

Forty milliliters of whole blood sample was collected from all participants. The peripheral blood mononuclear cells (PBMCs) were separated by density gradient centrifugation and stored at −196°C until use. For all experiments, the cryopreserved PBMCs were revived, rested overnight at 37°C in 5% CO_2_ in RPMI with 10% fetal bovine serum, and then used for various assays.

### Ethics Statement

The study was reviewed and approved by the Institutional Ethics Review Board of ICMR-National AIDS Research Institute (NARI/EC/2017-20). The study participants provided their written informed consent to participate in this study.

### Assessment of Specificity of HIV-gp140 Protein Probe

**Figure 2:**
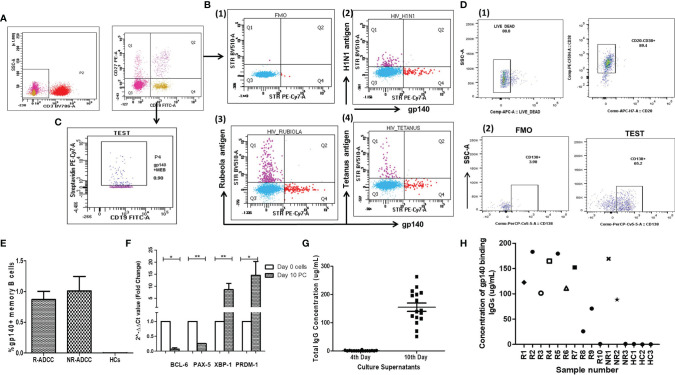
Generation of HIV-specific antibodies from HIV-specific memory B cells. **(A)** The dot plots show the gating strategy used to identify gp140+ memory B cells. The CD3− cells were gated from the lymphocytes which were then drilled down to gate CD3−CD19+CD27+ memory B cells. **(B)** To assess the binding specificity of the gp140 probe to a specific BCR, the PBMCs were co-stained with an HIV-gp140 protein probe and one of the three non-HIV antigen probes. The antigen-specific memory B cells were gated from CD3−CD19+CD27+ memory B cells. Subpanel **(B1)** represents the fluorescence minus one (FMO) control and subpanels **(B2-B4)** represent the dot plots showing the cells stained by biotinylated HIV-1 gp140 probe-conjugated with streptavidin PE-Cy7 (*X*-axis in all) and the streptavidin BV510 tagged biotinylated antigens, viz., H1N1 antigen, Rubeola antigen, and tetanus antigen, respectively (*Y*-axis). **(C)** From the CD3−CD19+CD27+ memory B cells, the gp140+ memory B cells were identified as CD3−CD19+CD27+gp140+ memory B cells. **(D)** The dot plots show the gating strategy used for plasma cells. 1) The live cells were gated from lymphocytes which were further drilled down to gate CD20−CD38+ plasmablast cells. 2) Further from these cells, plasma cells were gated as CD20−CD38+CD138+ cells (right panel) using FMO control. **(E)** The bar diagram depicts the frequencies of gp140-specific memory B cells (*Y*-axis) in R-ADCC LTNPs (*n* = 10), NR-ADCC LTNPs (*n* = 3), and HCs (*n* = 3) (*X*-axis) at the initiation of culture. **(F)** The cells at day 0 and day 10 of culture were analyzed for the expression of TFs of *BCL-6*, *PAX-5*, *XBP-1*, and *PRDM-1* genes using real-time PCR. The expression levels of mRNA were normalized with β-actin and fold change (ΔΔCT) (plotted on the *Y*-axis) was calculated with the expression of the respective genes at day 0 as baseline. The data represent the mean expression level from eight samples. The error bar indicates the standard error of the mean. **(G)** The scattered plot shows the concentrations of IgGs (μg/ml) (*Y*-axis) in the supernatants collected on the 4th and 10th day of culture (*X*-axis). **(H)** The concentration of gp140-binding antibodies (µg/ml) (*Y*-axis) generated from the gp140+ memory B cells of R-ADCC LTNPs (R1 to R10), NR-ADCC LTNPs (NR1 to NR3), and HIV-negative individuals (*X*-axis). The * represents P ≤ 0.05 and ** represents P ≤ 0.01.

We identified the HIV-Env-gp140-specific memory B cells using a biotinylated HIV-gp140 protein probe [HIV-1/clade B′/C (CN54)] (Immune Technology Corp. Lexington Ave., New York). Since the HIV envelope is also known to bind certain integrin and C-type lectin receptors expressed on B cells (22–24), the specificity of the gp140 probe was confirmed using unrelated antigen probes, viz., Influenza A H5N1 (A/chicken/India/NIV33487/06) Hemagglutinin/HA Protein (His Tag) (Sino Biological, Inc., China), Rubeola virus Native Protein (MyBioSource, USA), and tetanus toxoid C-fragment (TTCF) (MyBioSource, USA**)**. These probes were biotinylated using EZ-Link™ Sulfo-NHS-LC-Biotinylation Kit (Thermo Fisher Scientific, Waltham, MA, USA) as per the manufacturer’s protocol and tagged with streptavidin BV510 (BD Biosciences, CA, USA). The biotinylated gp140 probe was conjugated with streptavidin PE-Cy7 (BD Biosciences). The revived PBMCs from an LTNP were incubated with the gp140 protein probe and either of the unrelated antigen probes. After washing and fixating, the cells were acquired on BD FACSAria™ Fusion. The antigen-specific memory B cells were gated from CD3−CD19+CD27+ total memory B cells (**Figure 2A**). The quadrant was set on CD3−CD19+CD27 total memory B cells to gate respective antigen-specific memory B cells as shown in **Figure 2B**(1), (2), (3), and (4), respectively, for each unrelated antigen probe. The gating showed that the antigen-specific memory B cells were present in separate and mutually exclusive quadrants (Q1 and Q4), and the cells showing binding to both the probes (Q2 quadrant) were negligible (0.01%–0.02%), confirming the specificity of the HIV-gp140 probe.

### Identification and Sorting of HIV-gp140-Specific Memory B Cells

The HIV-specific memory B cells were identified using the HIV-gp140 protein probe as per the previously reported protocol (25, 26) with some modifications. In brief, after revival and resting, the PBMCs from the study participants were stained with CD3 BV786, CD19 FITC, and CD27 PE (all from BD Biosciences) and with biotinylated gp140 (5 µg/ml) for 30 min at room temperature and were then stained with streptavidin PE-Cy7 (BD Biosciences) for 30 min at room temperature and acquired on BD FACSAria™ Fusion (BD Biosciences). The total memory B cells were gated as CD3−CD19+CD27+ cells from lymphocytes (**Figure 2A**) which were further drilled down to identify HIV-gp140-specific memory B cells as CD3−CD19+CD27+gp140+ cells (**Figure 2C**). These cells were then sorted using BD FACSAria™ Fusion (BD Biosciences) and collected in the FACS tube. The purity of the sorted cells was >95% in all cases.

### Generation of Antibodies From gp140+ Memory B Cells

The sorted gp140-specific memory B cells (2 × 10^4^/ml) from all LTNPs (10 R-ADCC, 3 NR-ADCC LTNPs) and total memory B cells from three HCs were cultured in a 96-well plate for 10 days at 37°C and 5% CO_2_ in Iscove’s modified Dulbecco’s medium (Thermo Fisher) and 10% FBS along with human insulin (5 μg/ml) and human transferrin (50 μg/ml) (Sigma-Aldrich) and with different combinations of cytokines (detailed in **Supplementary Table 1**). On the 4th, 7th, and 10th day of culture, the supernatants were collected and stored at −20°C for further analysis. On the 10th day of culture, the cells were washed and stained for plasma cell markers: anti-human CD20 APC Cy7, CD38 PECF594, and CD138 PerCp Cy5·5 (BD Biosciences) and violet amine-reactive dye live-dead APC (Life Technologies) for 30 min at room temperature. The cells were acquired on BD FACSAria™ Fusion after fixing and analyzed by BD FlowJo (version 10.0) software. The gating strategy for the identification of plasma cells is depicted in **Figure 2D**.

### Expression of Transcription Factors Regulating Differentiation of Memory B Cells Into Plasma Cells

To confirm the differentiation of memory B cells into plasma cells, we assessed the expression of transcription factors (TFs) of *PAX-5*, *PRDM-1*, *BCL-6*, and *XBP-1* genes that regulate the differentiation of memory B cells using quantitative real-time PCR (RT-qPCR). The total RNA was extracted from cells at day 0 and day 10 culture using TRIzol^®^ Reagent method (Thermo Fisher) and quantitated using NanoDrop (Thermo Fisher). The cDNA was prepared using TaqMan^®^ Reverse Transcription Reagents (Thermo Fisher). The RT-qPCR was performed using *SY*BR^®^ Select Master Mix (Thermo Fisher) on ABI Thermal cycler HT 9700 (Applied Biosystems) according to the manufacturer’s protocol. The primer sequences are provided in **Supplementary Table 2**. The relative gene expression was normalized to *β-Actin* and calculated using the 2^−ΔΔCt^ method as described previously (27).

### Estimation of Total IgG in Supernatant

The 4- and 10-day culture supernatants were tested for the presence of IgG using Human IgG total Ready-SET-Go ELISA kit (Thermo Fisher Scientific) according to the manufacturer’s instructions. Briefly, the ELISA plate was coated overnight with purified anti-human IgG monoclonal antibody at 4°C. The next day, the wells were blocked with blocking buffer (1× PBS with 1% Tween 20 and 10% BSA) for 2 h at room temperature. All further incubations were carried out at room temperature. After washing, the culture supernatants (1:1,000 diluted) and 2-fold serially diluted IgG standards were added to the respective wells, and the plate was incubated for 2 h on a microplate shaker at 400 rpm. After washing, the horseradish peroxidase (HRP)-conjugated anti-human IgG antibody was added and the plate was incubated for 1 h, washed, and developed using 3,3′,5,5′-tetramethylbenzidine substrate (TMB) solution for 15 min. The reaction was then quenched using the stop solution (2 N H_2_SO_4_). The optical density (OD) was measured at 450 nm with a reference wavelength of 650 nm. The OD values of serially diluted standards were plotted on the *Y*-axis and their respective concentrations were plotted on the *X*-axis to create a graph to measure the IgG concentration from the culture supernatants based on their OD values.

### Purification of IgG From the Culture Supernatants

The IgG antibodies from the day 10 culture supernatants and the antibodies from the plasma of LTNPs showing ADCC activity were purified using NAb Protein G Spin Kit (Thermo Fisher) as per the manufacturer’s protocol and desalted using the Spin desalting columns (Thermo Scientific). The concentration was determined using NanoDrop (ND-1000). The concentration of antibodies purified from 10-day culture supernatants was found to be 40–80 μg/ml. The desalted purified IgG antibodies and their respective unbound fractions were stored at −20°C for further use.

### ELISA to Detect the Presence of HIV-gp140-Specific IgG Antibodies

The binding of purified antibodies (from 10 R-ADCC, 3 NR-ADCC LTNPs, and 3 HCs) to HIV-gp140 protein was assessed by gp140 ELISA as described previously (26). In brief, the high binding ELISA plate was coated overnight with 100 ng/well of purified gp140 protein at 4°C. Following washing with wash buffer (1× PBS with 0.05% Tween 20), the wells were blocked with the blocking buffer (1× PBS with 0.1% BSA and 1 mM EDTA) for 2 h. After washing, 50 µl of 4-fold serially diluted standards, i.e., anti-HIV immune globulin (HIVIG) (NIH AIDS Reagent Program) and purified antibodies (1 µg/ml), were added to the respective wells followed by incubation for 2 h at 37°C. The purified antibodies from the plasma of HIV-positive patients were used as a positive control (PC). The purified antibodies from the plasma of HIV-seronegative individuals were used as a negative control (NC) for the assay. The binding was then detected with the combination of HRP–anti-human IgG conjugate and TMB substrate. The OD values were measured at 450 nm with a reference wavelength of 650 nm. The cutoff value was calculated as (mean OD of PC + mean OD of NC)/6. The mean OD value of NC was 0.0685 and that of PC was 3.1965. The samples with OD values above the cutoff value of 0.544 were considered positive for gp140-binding antibodies. The OD values of diluted standards were plotted on the *Y*-axis and their respective concentrations were plotted on the *X*-axis to generate the reference graph. The concentrations of gp140-specific antibodies were determined by plotting the OD values on the reference graph.

### Rapid Fluorometric Antibody-Dependent Cellular Cytotoxicity Assay to Determine ADCC Activity of Generated Antibodies

**Figure 3:**
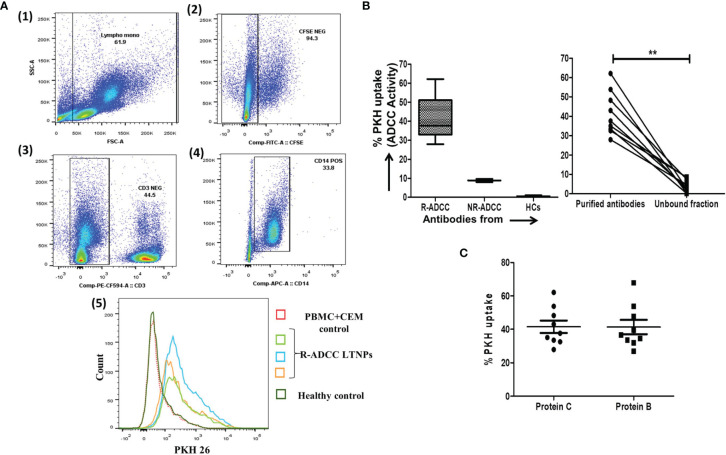
Assessment of ADCC activity of the generated antibodies. **(A)** The dot plots represent the gating strategy used for the RF-ADCC assay. The CFSE^neg^ cells (2) were gated from FSC/SSC scatters (1) which were drilled down to gate CD3− cells (3). The CD14+ monocytes (4) were then gated from CD3− cells and further analyzed for PKH expression. The histogram shows the comparison of PKH26 uptake by CD14**^+^** monocytes in the presence of purified antibodies generated from gp140-specific memory B cells from R-ADCC LTNPs (R2: green line, R4: blue line, and R5: orange line), HIV-seronegative (dotted line), and in PBMC+CEM control (red line) (5). **(B)** The box and whiskers plots (left panel) depict the ADCC activity (expressed in terms of % PKH uptake by monocytes) against HIV-1 Env C protein (left panel) (*Y*-axis) in R-ADCC (*n* = 10) and NR-ADCC (*n* = 3) LTNPs and HIV-negative individuals (*n* = 3) (*X*-axis). Boxes show median (IQR). The line diagram (right panel) shows the paired data of ADCC activity (*Y*-axis) of purified antibodies and their respective unbound fractions from R-ADCC LTNPs (*X*-axis) against HIV-1 Env C protein (right panel). **(C)** The scatter dot plot (lines showing the median with IQR) shows the ADCC activity (*Y*-axis) against Env C and Env B protein (*X*-axis) of purified antibodies generated from gp140-specific memory cells of R-ADCC LTNPs (*n* = 10). The Wilcoxon matched-pairs *t*-test was used for the data analysis and *p* ≥0.05 was considered to be significant for all the analyses. The ** represents P ≤ 0.01.

The generated purified antibodies showing gp140 specificity and their respective unbound fractions were tested for the ADCC against the Env-coated targets using rapid fluorometric antibody-dependent cellular cytotoxicity (RF-ADCC) assay as reported previously (28, 29). Briefly, the CEM.NKr-CCR5 target cells were coated with HIV-1 96ZM651 gp120 (HIV-1 C Env) and HIV-1 BaL gp120 (HIV-1 B Env) recombinant proteins (NIH AIDS Reagent Program) for 1 h at room temperature. Both coated and uncoated cells were then stained with the membrane dye PKH26 (Red Fluorescent Cell Linker, Sigma Aldrich, St. Louis, MO, USA) and carboxyfluorescein succinimidyl ester (CFSE) dye (Molecular Probes, UK) and incubated with purified antibody (2 µg/ml) or the unbound fraction at 37°C in 5% CO_2_ for 30 min. Subsequently, fresh PBMCs from a healthy donor were added as a source of effector cells in 1:10 target to effector ratio and incubated for 4 h at 37°C in 5% CO_2_ and then surface stained with CD3 PerCP and CD14 APCCy7 (both from BD Biosciences). The cells were acquired on BD FACSAria™ Fusion and analyzed using FlowJo software (version 10). The ADCC response was measured in terms of the uptake of the PKH26 dye from lysed target cells by CD3−CD14+ monocytes. The control of only effector cells + gp120-coated target was kept to check the background response. The gating strategy used for this assay is shown in **Figure 3A**.

### Reduction of Env-Reactivated Resting Memory CD4+ T Cells by the Generated ADCC Antibodies

**Figure 4:**
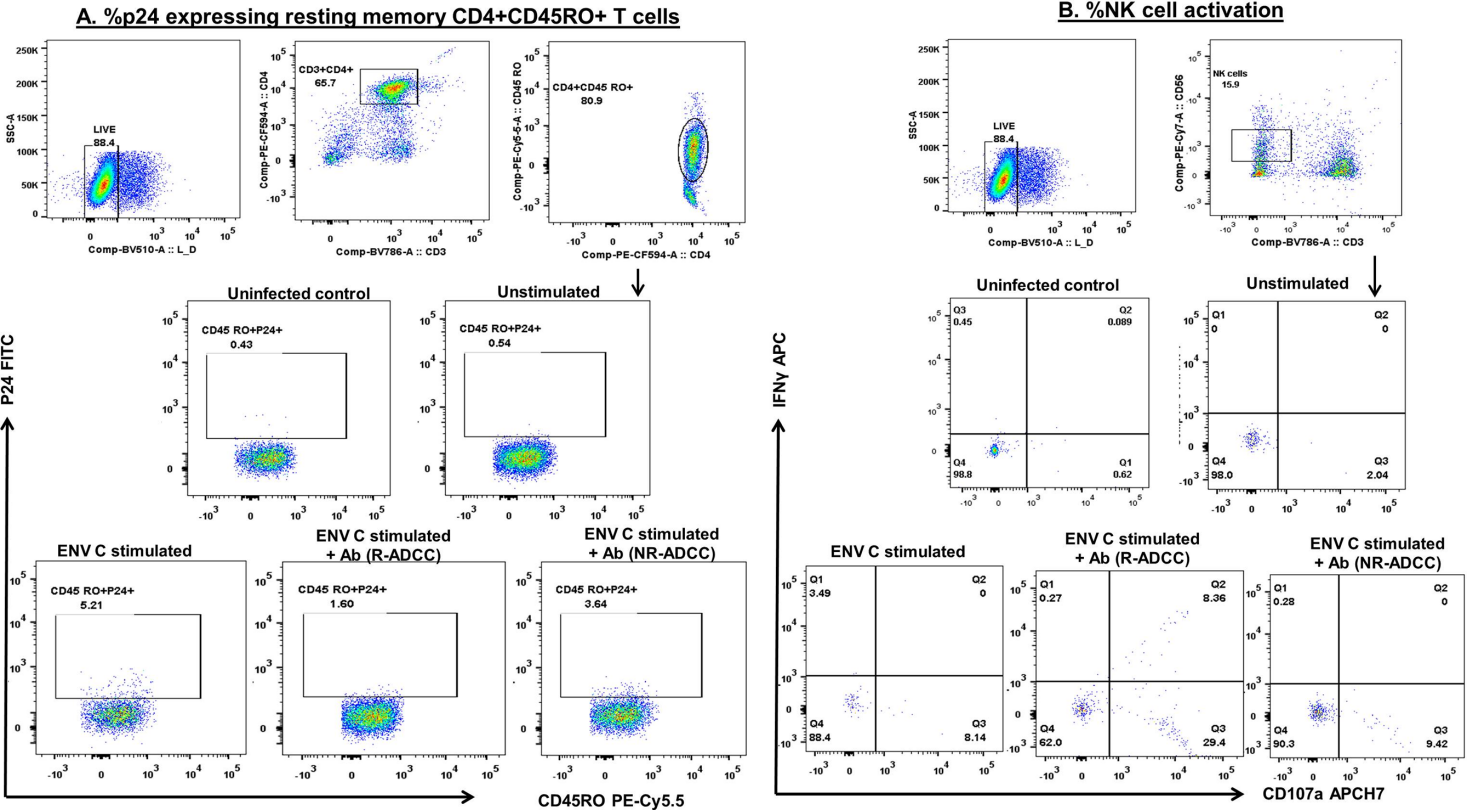
Gating strategy used to assess killing of reactivated HIV-1 latent reservoirs by the generated ADCC antibodies. To assess the ability of ADCC antibodies to lyse reactivated HIV latent reservoirs, the FACS-sorted resting memory CD4+ T cells (target) were reactivated with Env protein and co-cultured with CD4−CD8− cell (effector) in the presence of generated antibodies. The reduction in the frequencies of p24-expressing resting memory CD4+ T cells was used as the final readout of the assay. The dot plots depict the representative graphs for **(A)** the frequencies of p24-expressing resting memory CD4+ T cells. The live cells were gated from lymphocytes by using the live-dead dye. From the live cells, CD3+CD4+ cells were gated which were further drilled down to gate CD4+CD45RO+ resting memory T cells. These cells were then assessed for the expression of p24 in different culture conditions, viz., uninfected, unstimulated, Env-stimulated, and after the addition of antibodies from R-ADCC and NR-ADCC LTNPs. The uninfected cells were kept as a control to set the gate. **(B)** The dot plots show the gating strategy used to assess the simultaneous activation of CD3−CD56^dim^ NK cells (measured in terms of secretion of cytokine IFNγ and degranulation marker CD107a) in different culture conditions, viz., uninfected, unstimulated, Env-stimulated, and after the addition of IgGs from R-ADCC and NR-ADCC LTNPs.

The potential of *in-vitro*-generated ADCC antibodies to facilitate the killing of reactivated HIV-1 latent reservoirs was assessed in a flow-based latency reduction assay as used in our previous study (16). The reduction in the frequencies of intracellular p24-expressing resting memory CD4+ T cells was used as an indicator of the killing of reactivated HIV latent reservoirs by the NK cells in the presence of the generated purified antibodies. Briefly, CD4+CD45RO+ resting memory T cells (target) and CD4−CD8− effector cells (NK cells) were FACS sorted from the PBMCs of ART-naive HIV-infected individuals using CD4 PECF594, CD8 PE, and CD45 RO PEcy5.5 (all from BD Biosciences) as described previously (16). The sorted effector and target cells (1:1) were then incubated together in the presence of consensus HIV-1C envelope peptide pool (NIH AIDS Reagent Program) (5 µg/ml) for 3 h at 37°C in 5% CO_2_ and were then washed to remove peptides and incubated for 5 h with the antibodies generated from 5 R-ADCC and 2 NR-ADCC LTNPs in the presence of CD107a APCCy7 (BD Biosciences), brefeldin A (10 μg/ml) (Sigma), and monensin (0.68 μl/ml) (BD Biosciences) at 37°C in 5% CO_2_. Then, cells were surface stained with fixable viability stain 510, CD3 BV786, CD4 PECF594, CD45RO PE-Cy5.5, and CD56 PECy7 (all from BD Biosciences) for 30 min at room temperature. After permeabilization, the cells were stained for intracellular p24 (KC57-FITC) (Beckman Coulter, USA) and IFNγ APC (BD Biosciences) and incubated for 45 min at room temperature. After washing and fixing, the cells were acquired on BD FACSAria™ Fusion (BD Biosciences, USA) and analyzed using FlowJo software (Version 10). The gating strategy for identifying frequencies of p24-expressing cells in different experimental conditions is shown in **Figure 4A**. The gate for % p24-expressing cells was set using the stained uninfected cells. The percentages of p24 cells in unstimulated control (background response) were subtracted from the frequencies of p24+ cells in the respective Env-stimulated wells and used to calculate the percent p24 reduction using the formula: % p24 reduction = % p24+CD4+CD45RO+ cells in [(Env-stimulated well) − (Env+IgG well)]/(Env-stimulated well) × 100. Simultaneously, the activation (secretion of cytokine IFNγ and/or expression of degranulation marker CD107a) of CD3−CD56^dim^ NK cells was measured before and after the addition of purified antibodies as described previously (16) (**Figure 4B**).

### Comparison of the Potency of *In-Vitro*-Generated ADCC Antibodies With the Plasma ADCC Antibodies for the Killing of Reactivated Latent Reservoirs

Furthermore, we compared the potency of *in-vitro*-generated ADCC antibodies (from five R-ADCC LTNPs) to mediate the killing of reactivated latent reservoirs with the antibodies purified from the plasma samples of the same LTNPs.

### Statistical Analysis

The GraphPad Prism software (version 7.0) was used for statistical analyses. The Wilcoxon matched-pairs *t*-test was used to analyze the paired data of relative gene expression, ADCC activity, percent p24 expression, and NK cell activation. The comparison of the potency of generated antibodies with the plasma antibodies was performed using paired *t*-test. Throughout the text, the data are reported in the median (IQR) format.

## Results

### The HIV-Specific Memory B Cells Differentiated Into Plasma Cells at Day 10 of Culture

All LTNPs (R-ADCC and NR-ADCC) (median: 0.79%, IQR: 0.54%–1.225%), but not HCs, showed the presence of gp140-specific memory B cells (**Figure 2E**). We used a 10-day culture system representing the *in-vivo* antibody generation process to generate antibody-secreting plasma cells. On day 10, all cultures (10 R-ADCC and 3 NR-ADCC LTNPs and 3 healthy individuals) showed differentiation of memory B cells into CD20−CD38+ plasmablasts and CD20−CD38+CD138+ antibody-secreting plasma cells. The median frequency of plasmablasts was 80.2% (IQR: 75.5%–90%), and of these plasmablasts, 72.0% (IQR: 68.7%–82.2%) were CD20−CD38+CD138+ antibody-secreting plasma cells.

The transformation of memory B cells into plasma cells was also confirmed by assessing the upregulation of transcription factors required for plasma cell development. For that, the cells at day 0 and day 10 of cultures of eight samples (six R-ADCC and one NR-ADCC LTNPs and one HCs) were analyzed for the expression of four major transcription factors—*BCL-6*, *PAX-5*, *XBP-1*, and *PRDM-1*—that regulate memory B cell to plasma cell differentiation (30–32).

The expression of *PRDM-1* (*p* = 0.0078) and *XBP-1* (*p* = 0.0078) genes (required for the development and survival of plasma cells) was significantly increased in day 10 cells than day 0 cells. On the other hand, the expression of genes that maintain B-cell phenotype, i.e., *BCL-6* and *PAX-5*, was found to be decreased in day 10 cells than day 0 cells (*p* = 0.0142 and *p* = 0.0078, respectively) (**Figure 2F**), suggesting that memory B cells were successfully differentiated into plasma cells in the 10-day culture system.

### *In-Vitro*-Differentiated Plasma Cells Secreted IgG Antibodies

The culture supernatants of the 4th and 10th day of culture were tested for the presence of IgG. The 4-day culture supernatants of all the samples were negative for IgG (median: 0.00, IQR: 0.00–0.005), whereas the 10-day culture supernatants of all the samples showed the presence of IgG (OD: median: 0.253, IQR: 0.205–0.274). The median concentration of IgGs in the 10-day culture supernatants was 178.7 µg/ml (IQR: 133.9–211.8 µg/ml) (**Figure 2G**).

### The Antibodies Generated From Memory B Cells of LTNPs Showed Specificity to gp140

Of the 13 (10 R-ADCC and 3 NR-ADCC LTNPs) purified antibodies tested, 11 [(9 from R-ADCC (OD: range: median: 2.246, IQR: 1.684–3.014) and 2 from NR-ADCC (OD: 2.971 and 1.72)] showed specificity to gp140 protein. The purified antibodies generated from three HCs were used as a non-specific control. The concentration of gp140+ antibodies was 122.5 µg/ml (IQR: 88.52–169.4 µg/ml) with no difference in the concentration of gp140-specific antibodies from R-ADCC (median: 122.5 µg/ml, IQR: 86.16–172.2 µg/ml) and NR-ADCC (median: 129 µg/mL, IQR: 88.52–169.4) LTNPs (**Figure 2H**).

### Antibodies Generated From HIV-Specific Memory B Cells From R-ADCC LTNPs Showed Higher Potency to Mediate HIV-Specific ADCC

The antibodies showing specificity to gp140 (nine R-ADCC and two NR-ADCC LTNPs) along with the antibodies from three HCs as background control were tested for their ability to mediate HIV-Env C-specific ADCC by the RF-ADCC assay. No background ADCC activity was observed. The ADCC activity shown by the nine antibodies generated from R-ADCC LTNPs (median: 37.6%, IQR: 32.95%–51%, range: 27.9–62.1) was higher as compared to antibodies generated from two NR-ADCC LTNPs (median: 8.85%, IQR, range: 8%–9.7%) (**Figure 3B**, left panel) (**Table 1**) indicating that gp140-specific memory B cells from LTNPs with plasma ADCC could generate potent gp140-specific ADCC antibodies. Also, the ADCC was observed only with the antibody fraction in all samples (median: 37.6%, IQR: 32.95–51, range: 27.9–62.1) as compared with their respective unbound fraction (median: 3.0%, IQR: 1.05%–7.9%, range: 0.2–8.6) (*p* < 0.0001) (**Figure 3B**, right panel) indicating that cytotoxic activity was linked to the antibodies only.

**Table 1:** Characteristics of *in-vitro*-generated HIV-specific antibodies.

Sample ID		ADCC activity (% PKH26 uptake)		Latency reduction	
		Env C	Env B	% p24 reduction	% NK cell activation
**R1**		33.4	34.4	64.99	23.67
**R2**		35	36.5	Not assessed	
**R3**		43	31.9	62.50	50.27
**R4**		32.5	26.9	64.86	26.23
**R5**		27.9	53.8	Not assessed	
**R6**		53.8	47.7	61.05	21.56
**R7**		48.2	33.4	56.37	31.05
**R8**		37.6	39.5	Not assessed	
**R9**		62.1	67.8		
	Median	37.6	36.5	62.5	26.23
	IQR	32.95–51	32.65–50.75	58.71–64.92	22.62–40.66
	Range	27.9–62.1	26.9–67.8	56.37–64.99	21.56–50.27
**NR1**		8	5.9	9.86	7.14
**NR2**		9.7	2.5	17.81	13.15
	Median	8.85	4.2	13.84	10.15

The table shows the characteristics of the in-vitro-generated antibodies, viz., ADCC activity (% PKH26 uptake) against Env C and Env B protein and the killing of Env-reactivated resting memory CD4+ T cells (% p24 reduction and simultaneous % NK cell activation). The percentages of NK cell activation in unstimulated control (background response) were subtracted from the frequencies of NK cell activation after the addition of antibodies to the respective Env-stimulated wells. “R” represents the antibodies generated from R-ADCC LTNPs, i.e., the individuals with plasma ADCC activity, and “NR” represents the antibodies generated from NR-ADCC LTNPs, i.e., the individuals with no plasma ADCC activity. The number corresponds to each LTNP. The median, interquartile range (IQR), and range values are mentioned for both R and NR antibodies.

### The Antibodies Generated From R-ADCC LTNPs Showed Cross-Clade Activity

The cross-clade activity would be an important characteristic of the generated antibodies in their probable use as immune therapy. The antibodies generated from R-ADCC LTNPs showed similar activity against both Env C (median: 37.6%, IQR: 32.95%–51%, range: 27.9–62.1) and Env B (median: 36.5%, IQR: 32.65%–50.75%, range: 26.9–67.8) (**Figure 3C**; **Table 1**) indicating the cross-clade nature of these antibodies.

### The ADCC-Mediating Antibodies Also Showed the Ability to Initiate the NK Cell-Mediated Killing of Reactivated Latent HIV-1 Reservoirs

**Figure 5:**
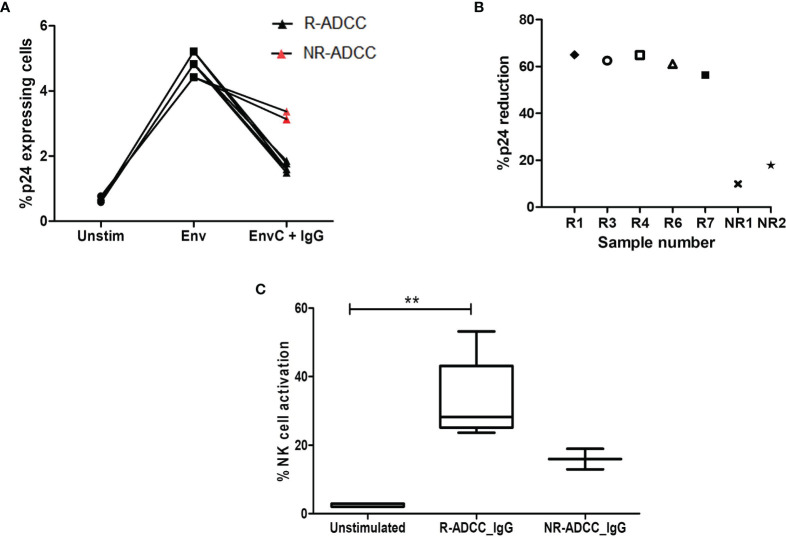
Assessment of killing of reactivated HIV-1 latent reservoirs by the generated ADCC antibodies. **(A)** The line diagram shows the % p24-expressing CD4+CD45RO+ resting memory T cells (*Y*-axis) in unstimulated cells, after Env C stimulation and after the addition of antibodies generated from R-ADCC LTNPs (black dots) and NR-ADCC LTNPs (red dots) to the Env-stimulated cells (*X*-axis). **(B)** The scatter plot shows % p24 reduction (*Y*-axis) in five R-ADCC LTNPs (R1, R3, R4, R6, and R7) and 2 NR-ADCC LTNPs (NR1 and NR2) (*X*-axis). **(C)** The box and whiskers plots % NK cell activation (*Y*-axis) in unstimulated cells, after Env C stimulation and after the addition of antibodies generated from R-ADCC LTNPs and NR-ADCC LTNPs to the Env-stimulated cells (*X*-axis). The Wilcoxon matched-pairs *t*-test was used to analyze the data and *p* ≥0.05 was considered to be significant. The ** represents P ≤ 0.01.

A flow-based latency reduction assay was used to determine the reduction in the percentages of Env C-reactivated p24-expressing CD4+ memory cells in the presence of generated antibodies and effector cells. Stimulation with HIV-1 Env C significantly increased the frequencies of p24+CD4+CD45RO+ memory cells (median: 4.83%, IQR: 4.42%–5.21%) as compared to the unstimulated cells (median: 0.64%, IQR: 0.59%–0.77%) (*p* = 0.0211) indicating the reactivation of resting memory CD4+ T cells. These frequencies were significantly reduced when antibodies generated from R-ADCC LTNPs (*n* = 5) were added (median: 1.6%, IQR: 1.54%–1.815%) (*p* = 0.0109), whereas marginal reduction in p24-expressing Env-stimulated cells was observed when antibodies generated from NR-ADCC LTNPs (*n* = 2) (median: 3.25%, IQR: 3.13%–3.37%) were added (**Figure 5A**). The percent p24 reduction of 62.5% (IQR: 58.71%–64.92%, range: 56.37–64.99) observed in the case of antibodies generated from R-ADCC LTNPs was 4.5-fold higher as compared to the reduction observed in the case of antibodies generated from NR-ADCC LTNPs (median: 13.84%, IQR, range: 9.863%–17.81%) (**Figure 5B**; **Table 1**).

Simultaneously, the percent NK cell activation (measured as IFNγ secretion and CD107 expression) was also increased significantly after the addition of antibodies generated from R-ADCC LTNPs (median: 28.27%, IQR: 25.11%–43.15%) as compared to the respective unstimulated control (median: 2.04%, IQR: 2.04%–2.94%) (*p* = 0.0041). However, the frequencies of activated NK cells after the addition of antibodies generated from two NR-ADCC LTNPs were increased marginally than their respective unstimulated controls (5.8% versus 12.94% and 5.8% versus 18.95%) (**Figure 5C**). Taken together, these observations indicate that HIV-specific antibodies generated from LTNPs with plasma ADCC activity showed higher potency to mediate ADCC and to effectively lyse the reactivated latent reservoirs as compared with the antibodies generated from the LTNPs without plasma ADCC activity.

The characteristics of the individual antibodies are compiled in **Table 1**. It was observed that the HIV-specific antibodies generated from R-ADCC LTNPs (R1 to R9) showed higher potency to mediate ADCC activity and also showed cross-clade ADCC activity as compared with the antibodies generated from NR-ADCC LTNPs (NR1 and NR2). Five of the nine antibodies generated from R-ADCC LTNPs (R1, R3, R4, R6, and R7) and antibodies generated from both NR-ADCC LTNPs (NR1 and NR2) were tested for their ability to lyse reactivated latent reservoirs. Four antibodies (R2, R5, R8, and R9) could not be tested in latency reduction assay due to the limitation of sample volume. All the five antibodies (R1, R3, R4, R6, and R7) showed higher potency to lyse Env-reactivated resting memory CD4+ T cells (% p24 reduction: median: 62.5%, IQR: 58.71%–64.92%, range: 56.37–64.99) as compared with the antibodies generated from NR-ADCC LTNPs (% p24 reduction: median: 13.84%, IQR/range: 9.863%–17.81%). Also, the simultaneous NK cell activation observed was higher in R-ADCC LTNPs (median: 26.23, IQR: 22.62–40.66, range: 21.56–50.27) than NR-ADCC LTNPs (median: 10.15, IQR, range: 7.14–13.15) (**Table 1**).

### *In-Vitro*-Generated ADCC Antibodies Showed Higher Potency Than the Plasma ADCC Antibodies

**Figure 6:**
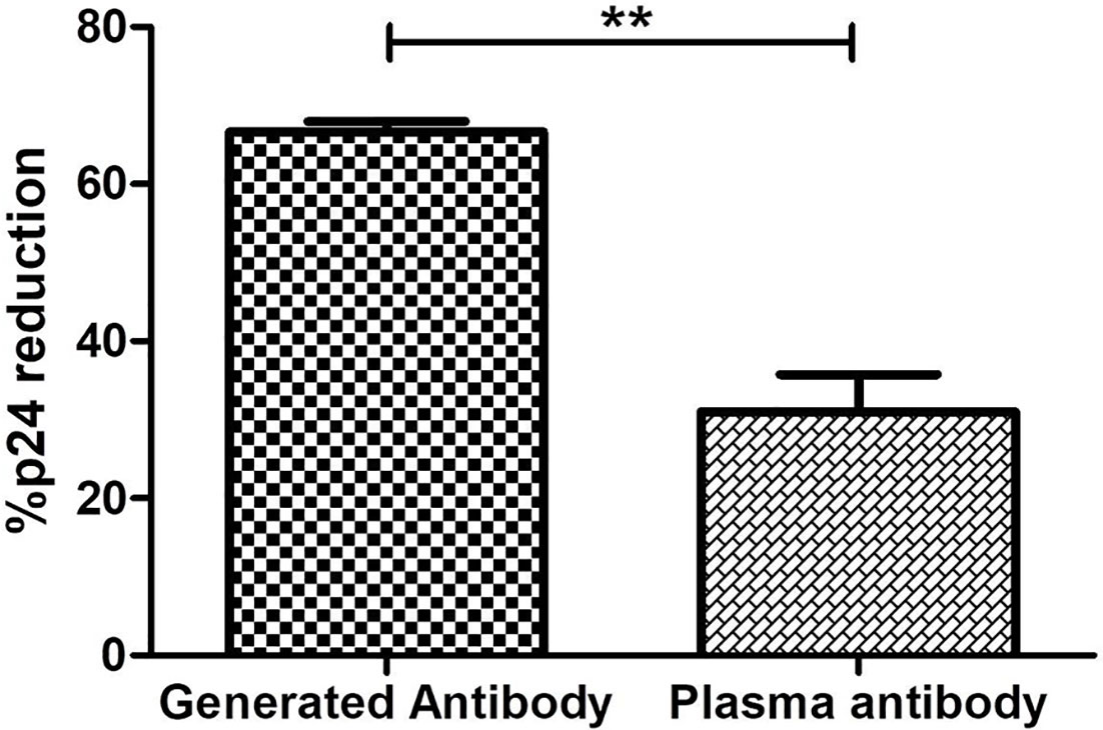
Comparison of potency to reduce latent reservoirs of generated antibodies and paired plasma antibodies. The bar diagram represents the comparative analysis of reduction in percent p24-expressing resting memory CD4+ T cells in the presence of antibodies generated from R-ADCC (*n* = 5) LTNPs and purified plasma antibodies of the same LTNPs (*n* = 5) (paired *t*-test, *p* = 0.0016). The ** represents P ≤ 0.01.

The *in-vitro*-generated antibodies should have good potency. The potency to mediate lysing of reactivated latent reservoirs of the generated ADCC antibodies and the antibodies purified from the plasma of the corresponding LTNPs was compared. The potency of the generated antibodies to lyse the reactivated reservoir (median: 62.5%, IQR: 58.71%–64.92%) was two-fold higher than the antibodies from the plasma of the respective ADCC responder LTNPs (median: 31.93, IQR: 21.33–40.37) (*p* = 0.0016) (**Figure 6**).

## Discussion

We attempted to generate ADCC-mediating antibodies and assessed their potency to assist in NK cell-mediated lysis of reactivated HIV reservoirs. By using the gp140-specific memory B cells of the LTNPs with and without plasma ADCC activity, we identified nine antibodies with potent ADCC activity, five of which were tested for their ability to facilitate NK cell-mediated lysis of Env-reactivated latent reservoirs. The study showed that only memory B cells from LTNPs with plasma ADCC could generate potent ADCC antibodies indicating the necessity of screening HIV-infected individuals for plasma ADCC before using memory B cells for antibody generation.

We used the streptavidin-conjugated biotinylated HIV-gp140 probe to sort the HIV-gp140-specific memory B cells. The range of the frequencies of memory B cells observed in our study [median: 0.79% (IQR: 0.54%–1.225%), **Figure 2E**] was similar with the frequencies reported in various studies using either the gp140 probe (33, 34) or the virus-like particles (35) indicating that the use of streptavidin-conjugated biotinylated gp140 probe for identification and further processing of memory B cells would be a useful strategy. The RF-ADCC assay used to measure the ADCC activity of generated antibodies in the present study has been reported to correlate with protection in human (36, 37) and animal (38, 39) studies. Hence, the ADCC activity shown by the generated antibodies might be relevant for its proposed use in protection and therapy.

The genetic variation within the envelope glycoprotein of different HIV-1 subtypes has been reported to be around 25% (40, 41). Hence, the generated antibodies must be able to elicit cross-clade ADCC to get a broad preventive or therapeutic response. The cross-clade nature of the antibodies generated in the present study signifies the importance of these antibodies in vaccine research.

The specificity of generated antibodies toward gp140 (comprising gp120 and gp41) indicates that these antibodies might be recognizing different epitopes of gp120 and gp41 proteins. Further studies to assess the epitope specificity of these antibodies will be useful in designing vaccines aiming at eliciting ADCC responses similar to the responses measured in LTNPs and ECs (6, 7).

The flow-based latency reduction assay used in the present study can simultaneously measure the reduction in p24-expressing reactivated reservoir and NK cell activation. Our laboratory (16) and others (15) previously used this short stimulation latency reduction assay. Using this assay, we have shown that the antibodies generated from R-ADCC LTNPs showed a remarkable reduction in reactivated latent reservoirs as compared with the antibodies generated from the NR-ADCC LTNPs, although the level of ADCC activity of individual antibodies generated from these LTNPs did not correlate with their ability to mediate latency reduction. Also, we have found that the generated ADCC antibodies could efficiently facilitate the lysis of the reactivated reservoir with two times higher potency than the plasma ADCC antibodies from the same population (**Figure 6**). These findings underscore important points: 1) The LTNPs were ART naive, and still, the potency of ADCC-mediating antibodies in circulation was lower; and 2) the HIV-infected individuals when they are on ART would be expected to have still lower plasma ADCC activity than LTNPs (17). These points indicate the importance of the use of the generated antibodies as the immune therapy to reduce the latent reservoir instead of relying upon the plasma ADCC antibodies. The presence of the compromised immune system in HIV infection and the possibility of undesired switching of an isotype that might happen while the preexisting B-cell repertoire is being activated (42, 43) might pose a hurdle in getting the desired reduction in latent reservoir using the shock and kill approach. Hence, the passive transfer of ADCC-mediating antibodies might be a promising strategy to achieve a reduction in the latent reservoir using the shock and kill approach. Additionally, this assay could also be useful for screening the latency reversal agents for their ability to reactivate the reservoir and also to stimulate the anti-HIV immune response.

The study had a few limitations also. Due to the sample limitation, we could not determine the HIV-neutralizing ability of these generated antibodies which could have been an added advantage for further research on these antibodies. The second limitation was the quantity of the antibodies available for further characterization. To obtain sufficient quantities for further characterization, the approach used in this study to identify and differentiate HIV-specific memory B cells could be used to screen the candidate plasma cells for the cloning of Ig variable regions to produce monoclonal antibodies in bulk. These antibodies then can be tested further for ADCC activity, virus neutralization, and lysis of reactivated HIV latent reservoirs. Also, the differentiated plasma cells can be further used to develop monoclonal antibodies using hybridoma technology, which is still a favored method, as it maintains the cognate pairing information of the generated antibodies and preserves the function of immune cells (44).

The ADCC involves both the antibodies and effector cells like NK cells. It is known that HIV-infected individuals are known to have compromised NK cell function (45). Although ADCC antibodies can effectively identify HIV-1-infected cells, the NK cells in these patients may not be able to lyse the reactivated reservoir (46). Hence, consideration of immune modulation by adaptive NK cell therapy along with the passive transfer of ADCC-mediating antibodies should be considered in HIV infection.

In conclusion, our study showed that a very low frequency of HIV-specific memory B-cell population can be sorted using the HIV-gp140 probe and cultured to effectively differentiate into plasma cells secreting HIV-specific ADCC antibodies showing cross-clade activity. The ability of these antibodies to facilitate NK cell-mediated lysis of reactivated latently infected cells indicates the importance of these antibodies in functional cure research. The mixture of monoclonal antibodies with multiple specificities generated using single-cell genomics would be important to explore for the polyclonal activities. The availability of such antibodies might be useful in studies aimed at the functional cure of HIV infection.

## Data Availability Statement

The original contributions presented in the study are included in the article/**Supplementary Material**. Further inquiries can be directed to the corresponding author.

## Ethics Statement

The studies involving human participants were reviewed and approved by the Institutional Ethics Review Board of ICMR-National AIDS Research Institute (NARI/EC/2017-20). The patients/participants provided their written informed consent to participate in this study.

## Author Contributions

MT: planning, monitoring the execution of the study, data analysis, and manuscript preparation. JD: planning, conducting the experiments, data analyses, and manuscript preparation. RB: counseling and enrollment of the study participants and manuscript preparation. All authors contributed to the article and approved the submitted version.

## Funding

This work was supported by the institutional funds of ICMR-National AIDS Research Institute, Pune, Government of India.

## Conflict of Interest

The authors declare that the research was conducted in the absence of any commercial or financial relationships that could be construed as a potential conflict of interest.

## Publisher’s Note

All claims expressed in this article are solely those of the authors and do not necessarily represent those of their affiliated organizations, or those of the publisher, the editors and the reviewers. Any product that may be evaluated in this article, or claim that may be made by its manufacturer, is not guaranteed or endorsed by the publisher.
