# Supplementary material for: HIV-gp140-Specific Antibodies Generated From Indian Long-Term Non-Progressors Mediate Potent ADCC Activity and Effectively Lyse Reactivated HIV Reservoir
Source: Front Immunol. 2022 Mar 2;13:844610. doi: 10.3389/fimmu.2022.844610 (PMC8924355; doi:10.3389/fimmu.2022.844610)
Supplement: Supplementary file 1 [file Table_1.docx]

| Culture Day | Culture conditions | Manufacturer |
| --- | --- | --- |
| 0^th^ Day | Phosphorothioate CpG oligodeoxynucleotide 2006 (ODN) (10ug/mL) | R&D Systems |
|  | Histidine tagged soluble recombinant human CD40L (5ug/mL) |  |
|  | Anti-polyhistidine mAb (5ug/mL) |  |
|  | IL-2 (20 U/mL) | BD Biosciences |
|  | IL-10 (50 ng/mL) |  |
|  | IL-15 (10 ng/mL) |  |
|  | IL-21 (100 ng/ml) |  |
| 4^th^ Day | IL-2 (20 U/mL) | R&D Systems |
|  | IL-6 (50 ng/mL) |  |
|  | IL-10 (50 ng/mL | BD Biosciences |
|  | IL-15 (10 ng/mL) |  |
| 7^th^ Day | IL-6 (50 ng/mL) | R&D Systems |
|  | IL-15 (10 ng/mL) | BD Biosciences |
|  | IFN-α (500 U/mL) | R&D Systems |

**Supplementary table 1:** Conditions for culturing HIV-specific memory B cells: The table shows the different stimulants and cytokines used for culturing of HIV-specific memory B cells at day 0, day 7 and day 10 of the in-vitro culture. IL: Interleukin, IFN: Interferon.
