# Supplementary material for: HIV-gp140-Specific Antibodies Generated From Indian Long-Term Non-Progressors Mediate Potent ADCC Activity and Effectively Lyse Reactivated HIV Reservoir
Source: Front Immunol. 2022 Mar 2;13:844610. doi: 10.3389/fimmu.2022.844610 (PMC8924355; doi:10.3389/fimmu.2022.844610)
Supplement: Supplementary file 2 [file Table_2.docx]

| **S. No** | **Oligo Name** | **Sequence (5' - 3')** |
| --- | --- | --- |
| 1 | Hu BCL-6: Forward-Q | CCTGCAGATGGAGCATGTTG |
| 2 | Hu BCL-6: Reverse-Q | CATCAGCATCCGGCTGTTG |
| 3 | Hu IRF4: Forward-Q | GCCAAGATTCCAGGTGACTC |
| 4 | Hu IRF4: Reverse-Q | ATCGTAGCCCCTCAGGAAAT |
| 5 | Hu PAX5: Forward-Q | CAGCAGGACAGGACATGGAG |
| 6 | Hu PAX5: Reverse-Q | CCTTGATGAGCAAGTTCCACT |
| 7 | Hu PRDM1: Forward-Q | ATGCGGATATGACTCTGTGGA |
| 8 | Hu PRDM1: Reverse-Q | CTGAACCGAAGTACCGCCATC |
| 9 | Hu XBP1: Forward-Q | AGTCCGCAGCAGGTGCAGGCCCA |
| 10 | Hu XBP1: Reverse-Q | ACTGGGTCCAAGTTGTCCAG |

**Supplementary table 2:** List of primers used for real time quantitative PCR analysis: The table represents the forward and reverse primer sets used for the real time qPCR analysis of expression of transcription factors PAX-5, PRDM-1, BCL6-6 and XBP-1 genes that regulate memory B cell to plasma cell differentiation. BCL6: B cell leukemia/lymphoma 6, IRF4: [interferon regulatory factor 4](https://www.ncbi.nlm.nih.gov/gene/3662), PAX5: [paired box 5](https://www.ncbi.nlm.nih.gov/gene/5079), PRDM1: PR/SET Domain 1, XBP1: X-Box Binding Protein 1.
